# Supplementary material for: A study of the effects of job insecurity on organizational citizenship behavior based on the chained mediating effects of emotional exhaustion and organizational identification
Source: PLoS One. 2025 Sep 8;20(9):e0329976. doi: 10.1371/journal.pone.0329976 (PMC12416719; doi:10.1371/journal.pone.0329976)
Supplement: S1 File — (DOCX) [file pone.0329976.s001.docx]

Attachment 1

| Table1 Mediation Effect Model Test (n=330) | | | | | | | | |
| --- | --- | --- | --- | --- | --- | --- | --- | --- |
|  | Emotional Exhaustion | | Organizational Identification | | Organizational Citizenship Behavior | | Organizational Citizenship Behavior | |
|  | *B* | *t* | *B* | *t* | *B* | *t* | *B* | *t* |
| Constants | 0.15 | 0.524 | 0.055 | 0.189 | -0.16 | -0.536 | -0.096 | -0.373 |
| Gender | -0.057 | -0.652 | -0.166 | -1.875 | 0.001 | 0.009 | 0.004 | 0.055 |
| Age | -0.03 | -0.64 | 0.02 | 0.418 | 0.003 | 0.062 | -0.015 | -0.367 |
| Education | 0.023 | 0.538 | -0.006 | -0.129 | -0.067 | -1.47 | -0.054 | -1.378 |
| Years of work experience | 0.018 | 0.646 | 0.002 | 0.085 | -0.002 | -0.078 | 0.006 | 0.244 |
| Job title | -0.068 | -1.089 | 0.014 | 0.216 | 0.03 | 0.457 | -0.006 | -0.112 |
| Industry | -0.004 | -0.196 | 0.01 | 0.482 | 0.054* | 2.532 | 0.050** | 2.716 |
| Organization size | 0.046 | 1.554 | 0.033 | 1.117 | 0 | -0.004 | 0.016 | 0.6 |
| Work nature | -0.039 | -1.093 | 0.002 | 0.045 | 0.02 | 0.524 | 0 | -0.001 |
| Job Insecurity | 0.626** | 14.334 | -0.315** | -5.572 | -0.582** | -12.805 | -0.215** | -4.066 |
| Emotional Exhaustion |  |  | -0.375** | -6.634 |  |  | -0.420** | -7.823 |
| Organizational Identification |  |  |  |  |  |  | 0.191** | 3.819 |
| *R* 2 | 0.405 | | 0.395 | | 0.354 | | 0.52 | |
| *Adjusted R* 2 | 0.388 | | 0.376 | | 0.336 | | 0.504 | |
| *F* value | *24.219* | | *20.786* | | *19.521* | | *31.349* | |
| * *p*<0.05 ** *p*<0.01 | | | | | | | | |

Table2 Results of Parallel Mediation Effect Test

| Effects | Item | Effect | *t* | LLCI | ULCI |
| --- | --- | --- | --- | --- | --- |
| Direct effect | Job Insecurity⇒Organizational Citizenship Behavior | -0.215 | -4.066 | -0.318 | -0.111 |
| Indirect effect | Job Insecurity⇒Emotional Exhaustion | 0.626 | 14.334 | 0.540 | 0.711 |
|  | Job Insecurity⇒Organizational Identification | -0.315 | -5.572 | -0.426 | -0.204 |
|  | Emotional Exhaustion⇒Organizational Identification | -0.375 | -6.634 | -0.485 | -0.264 |
|  | Emotional Exhaustion⇒Organizational Citizenship Behavior | -0.420 | -7.823 | -0.526 | -0.315 |
|  | Organizational Identification⇒Organizational Citizenship Behavior | 0.191 | 3.819 | 0.093 | 0.288 |
| Total Effect | Job Insecurity⇒Organizational Citizenship Behavior | -0.582 | -12.805 | -0.671 | -0.493 |

| Table3 Analysis of Indirect Effects | | | | |
| --- | --- | --- | --- | --- |
| Item | Effect | Boot SE | BootLLCI | BootULCI |
| Job Insecurity⇒Emotional Exhaustion⇒Organizational Citizenship Behavior | -0.263 | 0.047 | -0.357 | -0.171 |
| Job Insecurity⇒Organizational Identification⇒Organizational Citizenship Behavior | -0.060 | 0.030 | -0.128 | -0.008 |
| Job Insecurity⇒Emotional Exhaustion⇒Organizational Identification⇒Organizational Citizenship Behavior | -0.045 | 0.022 | -0.092 | -0.006 |
| Note: BootLLCI refers to the lower limit of the 95% bootstrap confidence interval, BootULCI refers to the upper limit of the 95% bootstrap confidence interval, bootstrap type: percentile bootstrap method. | | | | |
