# Supplementary material for: A study of the effects of job insecurity on organizational citizenship behavior based on the chained mediating effects of emotional exhaustion and organizational identification
Source: PLoS One. 2025 Sep 8;20(9):e0329976. doi: 10.1371/journal.pone.0329976 (PMC12416719; doi:10.1371/journal.pone.0329976)
Supplement: S2 File — (DOCX) [file pone.0329976.s002.docx]

# Impact of Job Insecurity on Citizen Organizational Behavior (overall survey questionnaire)

Dear Sir/Madam:

Greetings! Thank you very much for your participation in this research study. The purpose of this questionnaire is to explore the mechanism of job insecurity on organizational citizenship behavior. Your participation is important to our research. This questionnaire is anonymous and all data will be used for academic research only, without disclosing your personal information.

Please answer based on your true feelings, there are no right or wrong answers. The questionnaire takes about 10-15 minutes to complete.

---

## Part I: Basic Information

Please tick the appropriate box.

1. Gender:

□ Male □ Female

2. Age:

□ Under 25 years old □ 26-35 years old □ 36-45 years old □ Over 46 years old

3. Academic qualifications:

□ High school and below □ Specialized □ Bachelor's □ Master's and above

4. Years of work:

□ less than 1 year □ 1-3 years □ 4-6 years □ 7-10 years □ more than 10 years

5. Position held:

□ Grassroots employees □ Grassroots managers □ Middle managers □ Senior managers

6. The industry in which it is located:

□ Manufacturing □ IT/Internet □ Finance □ Education/Research

□ Services □ Government/Public Service □ Other ________

7. Size of organization (number of employees):

□ less than 50 people □ 51-200 people □ 201-500 people □ 501-1000 people □ more than 1000 people

8. Nature of work:

□ Full-time □ Part-time □ Contract □ Intern □ Other ________

1. Please create your identity match code [fill in the blank] *

Hint: The format is "initials - last four digits of cell phone number".

Example: Wang Ming, cell phone number 5678, fill in "WM-5678".

## Part II: Scale items

Please evaluate and judge the following descriptions based on your actual feelings and experiences, and mark "○" on the number that best matches.

### A. Job insecurity scale

Evaluation criteria: 1=Never 2=No 3=Almost never 4=Sometimes 5=Often 6=Always

1. My job is precarious.

2. My job may change in the future.

3. My job is not permanent.

4. I am concerned about the possibility of being dismissed.

5. The thought of being dismissed from my job terrifies me.

### B. Emotional exhaustion scale

Evaluation criteria: 1=Strongly disagree 2=Disagree 3=Somewhat disagree 4=Not sure 5=Somewhat agree 6=Agree 7=Strongly agree

1. I feel emotionally drained by work.

2. I feel burned out by my work.

3. I feel exhausted at the thought of facing another day at work.

### C. Organizational identity scale

Evaluation criteria: 1=Strongly disagree 2=Disagree 3=Not sure 4=Agree 5=Strongly agree

1. When someone praises my organization, it feels like a personal compliment.

2. I am concerned about what others think of my organization.

3. When someone criticizes my organization, it feels like a personal insult.

4. When talking about my organization, I usually use the word "we" rather than "they".

5. The success of the organization is my success.

6. I would be embarrassed if the media criticized my organization.

### D. Organizational Citizenship Behavior Scale

Evaluation criteria: 1=Strongly disagree 2=Quite disagree 3=Disagree 4=Not sure 5=Agree 6=Quite agree 7=Strongly agree

#### Helping Behavior

1. I will help any employee who can't keep up with their work.

2. I am willing to share my strengths with other members of the unit.

3. I try to act as a mediator when other members of the unit disagree.

4. I take measures to avoid conflicts with other members of the unit to the best of my ability.

5. I am willing to spend time helping unit members who are experiencing problems in their work.

6. I will greet other members of the unit in advance before doing anything that may affect them.

7. I will encourage other members of the unit when they are depressed.

#### Civic Ethics

8. I offer constructive suggestions on how to improve the efficiency of the unit.

9. I am willing to express my views on what is best for the unit at the risk of resentment.

10. I actively attend and participate in team meetings.

---

**Thank you again for your participation and support! **
