# Supplementary material for: A study of the effects of job insecurity on organizational citizenship behavior based on the chained mediating effects of emotional exhaustion and organizational identification
Source: PLoS One. 2025 Sep 8;20(9):e0329976. doi: 10.1371/journal.pone.0329976 (PMC12416719; doi:10.1371/journal.pone.0329976)
Supplement: Attachment — Submitted filename: Response to Reviewers.docx [file pone.0329976.s003.docx]

**Response to Reviewers**

Dear Editor and Reviewers:

Thank you for your letter and for the reviewers' valuable comments on our manuscript titled “A Study of the Effects of Job Insecurity on Organizational Citizenship Behavior Based on the Chained Mediating Effects of Emotional Exhaustion and Organizational Identification” (Manuscript ID: PONE-D-25-21939). These comments are very helpful for revising and improving our paper and provide important guidance for our research. We have now correctly named and uploaded three separate files as required by the journal editorial office: Response to Reviewers, Revised Manuscript with Track Changes, and Manuscript. Proper authorship is also indicated in the manuscript. The data analysis results have been accurately reported within the text, and additional related analyses have been uploaded as supplementary files. The original data have been deposited in the public database figshare, with corresponding statements included both in the Methods section of the manuscript and in the final Data Availability statement. Below, the reviewers’ comments are presented in italics and have been numbered for clarity. Please note that our responses are given in regular font, with the word “Response” highlighted in green. Furthermore, all revisions and additions in the latest version of the manuscript have been made using Word’s “Track Changes” feature. Please refer to the detailed point-by-point responses to the reviewers’ comments and concerns below.

**Responses to the comments of Reviewer #1:**

***第一部分：***

***Originality: Does the paper contain new and significant information adequate to justify publication?***

***1: The current form of the abstract is not well written. Please do not report the statistic result in the abstract. Author should highlight the novelty and main aim of the study. Make sure it commences with a succinct statement of the primary aim of the study, highlighting the novelty it brings to the field. This should be followed by a concise description of the research methodology employed, including the analysis techniques utilized and details regarding the sample collection process. Finally, it should conclude with a brief summary of the key findings and the implications derived from them. It is essential to emphasize the unique contributions of this study. Only highlight the most significant findings and implications to maintain focus.***

**Response:**

We sincerely thank you for this clear and valuable advice. We agree that the previous abstract was not well-structured. To address your concerns, we have completely rewritten the abstract following a formal, four-part structure: Objectives, Methods, Results, and Conclusions.

Here is the newly structured abstract for your review:

**Objectives**

This study aimed to explore the influence mechanism of job insecurity on organizational citizenship behavior (OCB). Specifically, it sought to examine the chained mediating role of emotional exhaustion and organizational identification in this relationship.

**Methods**

A longitudinal time-lagged survey was conducted on 330 employees at two time points. The data were collected using established scales for job insecurity, emotional exhaustion, organizational identification, and OCB. Structural equation modeling (SEM) was used to test the hypothesized chained mediation model.

**Results**

The results confirmed that emotional exhaustion and organizational identification are crucial mediators. Three significant indirect pathways were identified: (1) a simple mediation path through emotional exhaustion; (2) a simple mediation path through organizational identification; and (3) a chained mediation path where job insecurity increased emotional exhaustion, which in turn decreased organizational identification, ultimately leading to lower OCB. Notably, emotional exhaustion emerged as the most dominant mediating mechanism.

**Conclusions**

This study reveals a complex mechanism through which job insecurity impacts OCB, highlighting a sequential process from emotional strain to cognitive detachment. These findings offer important theoretical insights for job stress models and provide practical guidance for organizations to mitigate the negative effects of job insecurity by addressing both employee well-being and their sense of belonging.

***2: The introduction lacks a clear structure and focus. It would benefit from a more coherent presentation to effectively introduce the study's objectives and significance. Please follow this suggested structure: (1) Summarize the existing issue together with its importance. (2) Explain how researchers should address the discovered areas that need further investigation. (3) The research gap must clearly relate to the existing issue. (4) The research benefits from critiquing these aspects that previous academic studies avoid. (5) Review relevant past studies. (6) Draw a contrast between this study and all related past research. (7) Clearly state the research objectives. (8) The paper details how it introduces beneficial concepts to academic literature. A properly organized introduction creates better clarity by establishing solid groundwork for the overall paper.***

**Response:**

We sincerely thank you for this valuable suggestion. We agree that the original introduction was not well-structured and lacked a clear focus. To address this critical issue, we have completely rewritten the Introduction section following the 8-point structure you kindly provided. The new Introduction now proceeds as follows:

(1) It begins by establishing job insecurity as a critical contemporary issue and highlights the importance of OCB for organizational success.

(2) It explains the need to investigate the underlying mechanisms of the job insecurity-OCB link.

(3) It clearly identifies the research gap: the lack of understanding of the chained mediating process involving both emotional exhaustion and organizational identification.

(4) It critiques previous studies for largely focusing on single-mediator models and avoiding more complex, sequential pathways.

(5 & 6) It briefly reviews and contrasts our study with past research, emphasizing our unique contribution of integrating COR and Social Identity theories to test a sequential model.

(7) It explicitly states the three main objectives of our study.

(8) It concludes by detailing how our study introduces a beneficial concept (the chained mediation model) to the literature and its practical implications.

We believe this new structure provides a much clearer, more logical, and compelling rationale for our study, setting a solid groundwork for the rest of the paper.

***3: The introduction fails to connect properly the research issue to the fundamental concepts of this study. The discussion needs to present all essential concepts in a straightforward manner. Authors only tend to cover these constructs without proper linkage with the issues.***

**Response:3**

Thank you for pointing out this weakness. In our revised manuscript, we have made significant efforts to better connect the research issue (the impact of job insecurity) with the fundamental concepts (emotional exhaustion, organizational identification, OCB). The newly structured Introduction now establishes these links in a more straightforward and logical manner. For example, we now explicitly state that job insecurity is a stressor that harms OCB, and the core of our research problem is to uncover the sequential psychological process (affective reaction -> cognitive evaluation -> behavioral outcome) that connects them. The detailed theoretical elaboration of how each concept is linked has been moved to the new "Theoretical Framework and Hypotheses Development" section, where each link is justified step-by-step with clear theoretical backing.

***4: The authors fail to define their research gap with clarity and supporting evidence. The authors need to present a precise identification of the unaddressed areas in past research. All necessary arguments should be presented to validate the immediate need for addressing this gap. Provide a clear reasoning that demonstrates the study’s connection to contemporary conditions.***

**Response:**

We appreciate this crucial feedback. We acknowledge that the research gap was not clearly articulated in the original version. In the revised Introduction, we now dedicate a specific paragraph to clearly defining the research gap. We explicitly state that while the direct effects and single mediations have been studied, the sequential, chained mediating role of emotional exhaustion and organizational identification represents a significant and unaddressed area. We argue that past research has overlooked the potential sequence where an affective reaction (exhaustion) precedes a cognitive-evaluative shift (identification), and we provide a clear rationale for why investigating this multi-step process is important for a more complete understanding. We believe the research gap is now precise, well-justified, and clearly connected to contemporary research needs.

***第二部分：***

***Relationship to Literature: Does the paper demonstrate an adequate understanding of the relevant literature in the field and cite an appropriate range of literature sources? Is any significant work ignored?***

***5:Should have a standalone section for underpinning theory. This is to explain how the theory supporting the proposed paths and model. To enhance the clarity and coherence of the research, I recommend the authors thoroughly explain how or any theory support the current study and each path. By elaborating on the theory's key concepts and demonstrating its relevance to the research topic, the authors can establish a stronger connection between the theoretical foundation and the study's objectives.***

***6: Section 1.1 to 1.4 should be parked under the heading of literature review.***

**Response to 5 & 6:**

Thank you for these two very helpful structural recommendations. We agree that a more structured presentation of the theoretical background and hypothesis development was necessary. To address both comments simultaneously, we have implemented a significant structural revision:

We have created a new, standalone section titled "2. Theoretical Framework and Hypotheses Development" which now appears after the Introduction.

We have moved the content from our former sections 1.1, 1.2, 1.3, and 1.4 into this new section, under corresponding subheadings (2.1, 2.2, 2.3, and 2.4).

Within this new section, we have taken care to explicitly state the underpinning theories (e.g., Conservation of Resources Theory, Social Identity Theory) for each proposed path. We now clearly explain how each theory supports the specific hypothesis being developed.

This new structure accomplishes two goals: (1) it creates the "standalone section for underpinning theory" you requested, and (2) it logically parks the detailed literature review and hypothesis derivation in one coherent place. This change also allowed us to make the main Introduction more concise and focused, as per your earlier comments. We are confident that this revised structure greatly enhances the clarity and coherence of our manuscript.

***第三部分：***

***Methodology: Is the paper's argument built on an appropriate base of theory, concepts, or other ideas? Has the research or equivalent intellectual work on which the paper is based been well designed? Are the methods employed appropriate?***

***7: The research methodology is not well-written and not clear. Should have two sub-sections: (1) Research procedure and samples and (2) research instruments.***

**Response:**

Thank you for this valuable suggestion. We have reorganized the methodology section to improve its structure and clarity. We merged previous content into a new sub-section titled **“3.1 Participants and Procedure”** and renamed the “Research tools” section to **“3.2 Measures”**. We have also retained **“3.3 Data Analysis”** as a separate sub-section because our chained mediation analysis is a key feature of our study, and a dedicated section allows for a clear explanation of this advanced technique. We believe this revised three-part structure (Participants and Procedure, Measures, Data Analysis) enhances the logical flow and readability of our methodology.

***8:The authors should discuss the generalizability and representativeness of their sample in relation to the target population. Authors need to clearly explain how the chosen sample is intended to be representative of and reflective of the larger population. Any strategies employed to ensure a diverse and inclusive sample should also be highlighted. This will increase the credibility of the research findings and help readers understand the extent to which the results can be generalized to the broader population.***

**Response:**

Thank you for this important point. Our target population is the general workforce in China. We employed a convenience sampling method, recruiting participants through collaborations with various companies and an online survey platform. This strategy aimed to create a diverse sample in terms of industry, age, and tenure, thereby enhancing its representativeness of urban professionals in China. We acknowledge that as a non-probability sample, its generalizability to the entire national workforce is limited. In the interest of manuscript conciseness, we have not included a detailed discussion on generalizability in the main text but appreciate the opportunity to clarify our sampling rationale and its implications here.

***9:It is essential to provide a clear explanation of a stratified convenience sampling technique used in the study, along with the rationale for its selection. Why these techniques? What do you mean stratified convenience sampling technique? Stratified sampling technique is a probability sampling technique whereas convenience sampling technique is a non-probability sampling technique. Please relook into this. Are you referring to quota sampling technique? The authors should describe how they utilized this sampling technique to select respondents for the survey, ensuring generalizability and representativeness towards the targeted population. By doing so, readers can better understand the methodological approach and the potential limitations associated with the sample selection.***

**Response:**

Thank you for highlighting this inconsistency. We sincerely apologize for the error. You are correct; “stratified convenience sampling” was an inaccurate term. The method we used was convenience sampling. We have corrected this term throughout the manuscript to accurately reflect our methodology.

***10:Any selection criteria?***

**Response:**

Thank you for the question. Yes, we did use selection criteria. We have now clarified in the **“3.1 Participants and Procedure”** section that beyond being full-time employees, we screened the data to ensure quality. Specifically, our manuscript now states: “...the questionnaire included attention-check questions **and we screened for invalid data (e.g., excessively short completion times or logical inconsistencies)...**”

***11:It is good to include control variables which could be a confounding variable. However, author should provide justification for the inclusion of these control variables.***

**Response:**

Thank you for this valuable advice. We agree that controlling for potential confounding variables is important. Based on prior literature suggesting their influence on work-related outcomes, we collected demographic data on gender, age, education, and work tenure. We have now re-analyzed our data including these variables as controls (the results are available in the supplementary materials and confirm our main findings).

***12:The procedure of data collection needs to be elaborated further. The authors should explain in more detail how they collected the data, how they approached the respondents, and how they identified participants for the survey study. This explanation should be reasonable and logical, avoiding exaggerations and providing a clear account of the steps taken in the data collection process.***

**Response:**

Thank you. We have revised the **“3.1 Participants and Procedure”** section to provide a more detailed and accurate account of our data collection process. We have specified the recruitment channels (collaboration with HR departments and an online platform) and clarified the exact procedure for matching participant data across three time points. The revised text now reads: “...participants were instructed to create and use the same unique anonymous identification code (e.g., first letter of name - last four digits of cell phone number) for each survey. The research team subsequently used these codes to manually match the responses.”

***13:Any pretest and pilot test conducted prior to full scale study? Why?***

**Response:**

Thank you for this important question. Yes, a pilot test was conducted with 30 employees prior to the main study. Since our instruments were translated from English to Chinese, the primary purpose of the pilot test was to verify the clarity, comprehensibility, and cultural appropriateness of the translated items, and to perform a preliminary check of their reliability. This step was crucial for ensuring the quality of our measurement tools. To maintain the conciseness of the manuscript, we have not detailed this step in the paper itself, but we confirm it was an integral part of our research design.

***14:Why SPSS PROCESS MACRO software was used in this study?***

**Response:**

Thank you for this question. We have added justification for our choice of software in the **“3.3 Data Analysis”** section. We used the SPSS PROCESS macro because it is specifically designed for testing complex mediation models like the chained mediation in our study (Model 6). Furthermore, its bootstrapping procedure is a statistically powerful method for testing indirect effects, as it does not assume a normal distribution of the sampling effect and provides more accurate confidence intervals, making it superior to traditional methods like the Sobel test for our research questions.

***15: The research instrument section is too lengthy. Please simplify it.***

**Response:**

Thank you for the feedback. We agree and have condensed the **“3.2 Measures”** section. For each scale, we removed superfluous theoretical descriptions and redundant concluding phrases. We have retained all the essential information needed for replication, including the scale's citation, number of items, response format, a brief description of the dimensions measured, and the Cronbach’s alpha from our study. We believe the section is now more concise and direct.

***第四部分***

***Results: Are results presented clearly and analysed appropriately? Do the conclusions adequately tie together the other elements of the paper?***

***16. The analysis results reporting is not clear. Author should report the preliminary analysis result (common method bias test, descriptive analysis results, and respondents profile characteristics), measurement model analysis (reliability, convergent validity, discriminant validity), and structural model analysis (path result).***

**Response:**

***第五部分***

***Practicality and/or Research implications: Does the paper identify clearly any implications for practice and/or further research? Are these implications consistent with the findings and conclusions of the***

***17.The structure of the conclusion part should be as follows: (1) Discussion, (2) Theoretical Implications, (3) Practical Implications, (4) Limitations and Future Research Recommendations, and (5) Conclusion.***

***19.Should have a standalone section for theoretical implication. This section should discuss the implications of the study's findings and how they contribute to the existing theoretical knowledge. Summarize the key findings and their relevance to the existing theoretical frameworks or models. Analyze how the findings align with or challenge current theoretical perspectives and concepts related to all the key concepts of this study. Discuss any theoretical insights or advancements that the study provides and highlight how the findings contribute to a deeper understanding of the research area.***

***20.Should have a standalone section for practical implication. The authors should provide valuable insights based on current practices and policies, supported by evidence from their research. To strengthen the practical implications, it is crucial to reference specific findings, data, or examples that demonstrate the validity and reliability of the recommendations. By incorporating this approach, the authors can offer concrete and actionable suggestions that have a solid grounding in their research findings.***

**Response 17,19&20:**

We sincerely thank the reviewer for these detailed and highly constructive comments regarding the manuscript's structure. We agree completely that a clear and logical delineation of the discussion, theoretical contributions, practical advice, and limitations is essential for maximizing the impact and readability of our work.

Guided by your valuable feedback, we have undertaken a significant restructuring of the paper's final sections. While we carefully considered creating five separate standalone sections, we found that integrating the core elements into a single, cohesive Section 4: Discussion created a more powerful and seamless narrative flow. We believe this approach allows the reader to immediately and logically connect our empirical findings with their direct theoretical significance and practical applications, without disrupting the argumentative thread.

To ensure that these distinct contributions are still clearly identifiable, as you rightly suggested, we have structured the new Discussion section thematically. It now begins with an in-depth analysis of the core findings, and is immediately followed by a distinct, dedicated paragraph on Theoretical Implications, another on Practical Implications, and concludes with a paragraph outlining the study's Limitations and Future Research. The Conclusion then follows as a separate and final Section 5.

***18.Discussion part should be presented more concise.***

**Response:**

We appreciate this valuable advice. In our revision, we have carefully edited the entire Discussion section to improve its conciseness. We have streamlined the presentation of our findings, eliminated redundant phrasing, and focused on making our analysis more direct and impactful. By structuring the section as a single, flowing narrative, we have also reduced the repetition that can sometimes occur when transitioning between multiple, separate sub-sections, resulting in a more succinct and focused presentation overall.

***21. A conclusion section is required with not more than 150 words.***

**Response:**

Thank you for this clear instruction. We have followed your recommendation and completely rewritten the conclusion. The new, standalone Section 5: Conclusion is now a concise summary of our study's key message and findings, and at 129 words, it strictly adheres to the requested 150-word limit.

***第六部分***

***Quality of Communication: Does the paper clearly express its case, measured against the technical language of the field and the expected knowledge of the journal's readership? Has attention been paid to the clarity of expression and readability, such as sentence structure, jargon use, acronyms, etc.***

***22.Proofread the manuscript, Clarity is required, and cite more recent relevant studies. Please also check citation format and referencing style.***

Thank you for your valuable feedback. We have thoroughly proofread the manuscript for clarity, incorporated more recent relevant research, and ensured all citations and references strictly adhere to the journal's formatting guidelines.

**Responses to the comments of Reviewer #2:**

***1.I recommend removing detailed numerical statistical findings from the abstract, as these may reduce clarity for general readers. Instead, consider presenting your key findings in a concise and accessible manner to ensure broader understanding.***

**Response:**

We sincerely thank you for this clear and valuable advice. We agree that the previous abstract was not well-structured. To address your concerns, we have completely rewritten the abstract following a formal, four-part structure: Objectives, Methods, Results, and Conclusions.

Here is the newly structured abstract for your review:

**Objectives**

This study aimed to explore the influence mechanism of job insecurity on organizational citizenship behavior (OCB). Specifically, it sought to examine the chained mediating role of emotional exhaustion and organizational identification in this relationship.

**Methods**

A longitudinal time-lagged survey was conducted on 330 employees at two time points. The data were collected using established scales for job insecurity, emotional exhaustion, organizational identification, and OCB. Structural equation modeling (SEM) was used to test the hypothesized chained mediation model.

**Results**

The results confirmed that emotional exhaustion and organizational identification are crucial mediators. Three significant indirect pathways were identified: (1) a simple mediation path through emotional exhaustion; (2) a simple mediation path through organizational identification; and (3) a chained mediation path where job insecurity increased emotional exhaustion, which in turn decreased organizational identification, ultimately leading to lower OCB. Notably, emotional exhaustion emerged as the most dominant mediating mechanism.

**Conclusions**

This study reveals a complex mechanism through which job insecurity impacts OCB, highlighting a sequential process from emotional strain to cognitive detachment. These findings offer important theoretical insights for job stress models and provide practical guidance for organizations to mitigate the negative effects of job insecurity by addressing both employee well-being and their sense of belonging.

***2.There appears to be inconsistency in how the term Conservation of Resources Theory is referenced, as it is also labeled at times as resource preservation theory or resource conservation theory. While this may stem from translation issues, ensuring consistent and accurate use of terminology will strengthen the manuscript’s academic clarity.***

**Response:**

Thank you for pointing out this inconsistency. We have carefully reviewed the manuscript and have standardized the term to the widely accepted "Conservation of Resources (COR) Theory" throughout the paper to ensure academic clarity and accuracy.

***3.It would enhance the manuscript’s impact to include dedicated sections for Theoretical Implications and Managerial Implications. These sections would clarify the study’s contributions to both academic discourse and practical application—potentially including recommendations for organizational practice or policy.***

**Response:**

Thank you for this valuable suggestion. We completely agree that explicitly discussing the theoretical and managerial implications is crucial for enhancing the manuscript's impact.

Following your advice, we have substantially revised the Discussion section. Specifically, we have dedicated the final two paragraphs of this section to thoroughly address these points. The first of these paragraphs now focuses exclusively on the Theoretical Implications of our findings, while the subsequent paragraph is dedicated to the Managerial Implications, offering practical recommendations.

We chose to integrate these discussions as distinct paragraphs at the end of the main Discussion section, rather than creating separate subsections, to better align with the common narrative style of manuscripts published in PLOS ONE. We believe this approach effectively highlights these critical contributions while maintaining a cohesive flow.
