# Supplementary material for: A study of the effects of job insecurity on organizational citizenship behavior based on the chained mediating effects of emotional exhaustion and organizational identification
Source: PLoS One. 2025 Sep 8;20(9):e0329976. doi: 10.1371/journal.pone.0329976 (PMC12416719; doi:10.1371/journal.pone.0329976)
Supplement: Attachment — Submitted filename: Response_to_Reviewers_auresp_2.docx [file pone.0329976.s004.docx]

**Response to Reviewers**

Dear Editor and Reviewers:

Thank you for your letter and for the reviewers' valuable comments on our manuscript titled “A Study of the Effects of Job Insecurity on Organizational Citizenship Behavior Based on the Chained Mediating Effects of Emotional Exhaustion and Organizational Identification” (Manuscript ID: PONE-D-25-21939). These comments are very helpful for revising and improving our paper and provide important guidance for our research. We have now correctly named and uploaded three separate files as required by the journal editorial office: **Response to Reviewers, Revised Manuscript with Track Changes, and Manuscript.** Proper authorship is also indicated in the manuscript. The data analysis results have been accurately reported within the text, and additional related analyses have been uploaded as supplementary files. The original data have been deposited in the public database figshare, with corresponding statements included both in the Methods section of the manuscript and in the final Data Availability statement. Below, the reviewers’ comments are presented in italics and have been numbered for clarity. Please note that our responses are given in regular font, with the word “Response” highlighted in green. Furthermore, all revisions and additions in the latest version of the manuscript have been made using Word’s “Track Changes” feature. Please refer to the detailed point-by-point responses to the reviewers’ comments and concerns below.

**Responses to the comments of Reviewer #1:**

We sincerely thank the reviewer for their positive evaluation and strong endorsement of our manuscript. We are greatly encouraged by their recognition of our research.

**Responses to the comments of Reviewer #2:**

1. Please adopt a single citation format throughout the manuscript (Vancouver style). I noticed instances of duplicate citations in mixed styles. For example:
   “The study employed the validated Job Insecurity Scale developed by Mauno et al. (2001)[30] to assess employees’ subjective perceptions of job stability.”
   **could be streamlined to:**
   “The study employed the validated Job Insecurity Scale to assess employees’ subjective perceptions of job stability [30].”

**Response:**

Thank you for bringing this formatting inconsistency to our attention. We have carefully reviewed the entire manuscript and standardized all citations to follow the Vancouver style consistently. Specifically, we have removed the author names and publication years from in-text citations, retaining only the numerical references in square brackets. The instances you identified in the methodology section have been corrected as follows:

1.“The study employed the validated Job Insecurity Scale developed by Mauno et al. (2001)[30]” has been revised to “The study employed the validated Job Insecurity Scale [30]”

2. Similar corrections have been applied to all other measurement instruments and analytical framework citations throughout the manuscript
3. We have conducted a comprehensive review to ensure no other formatting inconsistencies remain in the document.

2. Section 4.1 — Common Method Bias (CMB) test
The manuscript currently states:
“The 305 results of Harman’s single-factor test indicated that the unrotated first common factor accounted for 49.59 % of the variance, thereby indicating the presence of common method bias, which was insufficient to overshadow the study’s findings.”
Because 49.59% falls below the conventional 50% threshold, it should not be interpreted as evidence of common method variance (CMV). Nevertheless, the risk of CMV is reinforced by VIF values greater than 3.3 (Kock, 2015; <https://doi.org/10.4018/ijec.2015100101)>. I strongly recommend conducting an additional diagnostic—such as the Lindell & Whitney marker‐variable test (2001; <https://doi.org/10.1037/0021-9010.86.1.114>)—to determine whether CMB meaningfully inflates the correlations among your variables. For a comprehensive overview, see Podsakoff et al. (2023; <https://doi.org/10.1146/annurev-orgpsych-110721-040030).>

**Response:**

We are grateful for this very insightful comment regarding the Common Method Bias (CMB) analysis. We agree that our initial interpretation was not sufficiently nuanced and appreciate the valuable references provided. As we are in the advanced stages of revision and do not have the opportunity to collect new data to perform the suggested marker-variable test, we have taken a two-fold approach to address your concern, following your guidance:

**1.Revised Section 4.1:** To proactively mitigate common method bias (CMB), a three-wave, time-lagged research design was employed, which is a key procedural remedy. We further conducted statistical analyses to assess potential remaining bias. Harman's single-factor test revealed that the first unrotated factor accounted for 49.59% of the variance, falling below the 50% threshold. Additionally, a full collinearity assessment was performed, yielding a maximum variance inflation factor (VIF) of 3.36. While we acknowledge that this VIF value is slightly above the strict 3.3 benchmark suggested by Kock (2015), it remains well within other commonly accepted thresholds (e.g., 5 or 10). Considering our robust time-lagged design as the primary control, coupled with these statistical results, we conclude that common method bias is not a significant concern for this study.

**2.Added to Limitations:** We have also added a paragraph to the “Limitations and Future Research” section. In this paragraph, we explicitly state that the study did not employ the marker-variable technique and recommend that future research could benefit from incorporating this method for a more robust assessment of CMB. We believe these revisions transparently address the potential for CMB and align our manuscript with the rigorous standards you have suggested. The modified sections are provided below for your review.

3. To further underscore the relevance and urgency of your OCB investigation, you may wish to incorporate insights from recent literature, for example:
• <https://doi.org/10.1108/LODJ-08-2023-0433>
• <https://doi.org/10.1108/K-04-2024-1049>
• <https://doi.org/10.1177/21582440241268848>

**Response:**

Thank you for recommending these valuable contemporary references. We have strategically incorporated all three suggested papers into the end of the first paragraph of the introduction to strengthen our theoretical foundation and demonstrate the continued relevance of OCB research. The specific additions are as follows:

***“Recent empirical evidence has further underscored the critical role of OCB across diverse organizational contexts, demonstrating its enhancement through servant leadership and psychological empowerment [7], its significance in challenging institutional environments [8], and its complex relationship with individual characteristics requiring mediational pathways [9].”***
